# Mitochondria–Nucleus Shuttling FK506-Binding Protein 51 Interacts with TRAF Proteins and Facilitates the RIG-I-Like Receptor-Mediated Expression of Type I IFN

**DOI:** 10.1371/journal.pone.0095992

**Published:** 2014-05-01

**Authors:** Taishin Akiyama, Takuma Shiraishi, Junwen Qin, Hiroyasu Konno, Nobuko Akiyama, Miho Shinzawa, Maki Miyauchi, Nobukazu Takizawa, Hiromi Yanai, Hiroyuki Ohashi, Etsuko Miyamoto-Sato, Hiroshi Yanagawa, Weidong Yong, Weinian Shou, Jun-ichiro Inoue

**Affiliations:** 1 Division of Cellular and Molecular Biology, Institute of Medical Science, The University of Tokyo, Minato-ku, Tokyo, Japan; 2 Department of Developmental and Regenerative Biology, Key Laboratory for Regenerative Medicine, Ministry of Education and International Base of Collaboration for Science and Technology, the Ministry of Science and Technology and Guangdong Province, Jinan University, Guangzhou, China; 3 Division of Interactome Medical Sciences, Institute of Medical Science, The University of Tokyo, Minato-ku, Tokyo, Japan; 4 Department of Biosciences and Informatics, Faculty of Science and Technology, Keio University, Yokohama, Japan; 5 Pediatrics, Biochemistry and Molecular Biology, Medical and Molecular Genetics, Pharmacology and Toxicology, Wells Center for Pediatric Research, Indiana University School of Medicine, Indianapolis, Indiana, United States of America; Toho University School of Medicine, Japan

## Abstract

Virus-derived double-stranded RNAs (dsRNAs) are sensed in the cytosol by retinoic acid-inducible gene (RIG)-I-like receptors (RLRs). These induce the expression of type I IFN and proinflammatory cytokines through signaling pathways mediated by the mitochondrial antiviral signaling (MAVS) protein. TNF receptor-associated factor (TRAF) family proteins are reported to facilitate the RLR-dependent expression of type I IFN by interacting with MAVS. However, the precise regulatory mechanisms remain unclear. Here, we show the role of FK506-binding protein 51 (FKBP51) in regulating the dsRNA-dependent expression of type I IFN. The binding of FKBP51 to TRAF6 was first identified by “*in vitro* virus” selection and was subsequently confirmed with a coimmunoprecipitation assay in HEK293T cells. The TRAF-C domain of TRAF6 is required for its interaction, although FKBP51 does not contain the consensus motif for interaction with the TRAF-C domain. Besides TRAF6, we found that FKBP51 also interacts with TRAF3. The depletion of FKBP51 reduced the expression of type I IFN induced by dsRNA transfection or Newcastle disease virus infection in murine fibroblasts. Consistent with this, the FKBP51 depletion attenuated dsRNA-mediated phosphorylations of IRF3 and JNK and nuclear translocation of RelA. Interestingly, dsRNA stimulation promoted the accumulation of FKBP51 in the mitochondria. Moreover, the overexpression of FKBP51 inhibited RLR-dependent transcriptional activation, suggesting a scaffolding function for FKBP51 in the MAVS-mediated signaling pathway. Overall, we have demonstrated that FKBP51 interacts with TRAF proteins and facilitates the expression of type I IFN induced by cytosolic dsRNA. These findings suggest a novel role for FKBP51 in the innate immune response to viral infection.

## Introduction

Recognition of nonself nucleic acids is crucial for the initiation and modulation of the innate immune pathways in response to viral infection [1]–[4]. The double-stranded RNAs (dsRNAs) derived from some RNA and DNA viruses are recognized as pathogen-associated molecular patterns by the innate immune system. The retinoic acid-inducible gene-I (RIG-I)-like receptors (RLRs)–RIG-I, melanoma differentiation associated factor 5 (MDA-5), and laboratory of genetics and physiology 2 (LGP2)–function as cytosolic sensors of virus-derived dsRNAs [5]–[8]. RLRs that recognize dsRNAs activate the signaling pathways that drive the production of type I IFN, which induce antiviral responses by upregulating the expression of a wide variety of IFN-stimulated genes [1]–[4].

Although RIG-I and MDA-5 recognize different structures of dsRNAs and distinct viruses [6], the dsRNA sensing by both RLRs is transmitted to a common downstream regulator, mitochondrial antiviral signaling (MAVS) (also known as IPS-1, CARDIF, and VISA) [9]–[12]. The caspase recruit domain (CARD) of the RLRs interacts with the N-terminal CARD of MAVS. This CARD–CARD interaction initiates the formation of the multiprotein MAVS signaling complex [3], [13], [14], anchored to the mitochondria [9] and peroxisomes [15]. Formation of the MAVS signaling complex then leads to the activations of the IKK complex, containing IκB kinase α (IKKα), IKKβ, TNF receptor-associated factor (TRAF) family member-associated NF-κB activator-binding kinase (TBK1), and IKKε [14]. The activation of the IKK complex phosphorylates IκB, which sequesters NF-κB in the cytosol. Subsequently, the phosphorylation of IκBα triggers its proteasome-dependent degradation, which in turn causes the nuclear localization of NF-κB [16]. In contrast, the activation of the atypical IKKs (i.e., TBK1 and IKKε) promotes the phosphorylation, homodimerization, and nuclear localization of IFN regulatory factor 3 (IRF3) and IRF7 [17], [18]. The nuclear localization of these transcription factors promotes the transcription of the type I IFN and proinflammatory cytokines [19].

The TRAF family proteins transduce various signals leading to the activation of various transcription factors. Recent studies have suggested that the TRAF family proteins mediate the RLR signals as components of the MAVS complex [20]–[25]. The expression of type I IFN elicited by the infection of RNA viruses was severely reduced in TRAF3-deficient mouse embryonic fibroblast (MEF) cells, indicating a crucial role for TRAF3 in RLR-dependent signaling [20], [21]. We and another group have previously reported that the RLR-mediated expression of type I IFN was reduced in TRAF6-deficient MEF and conventional dendritic cells [22], [23]. Furthermore, MAVS was shown to interact with TRAF2, TRAF3, and TRAF6 through TRAF-binding consensus sequences [12], [21]. Consistent with this, mutations in these motifs impaired MAVS-mediated NF-κB activation and type I IFN production [12], [26]. These data suggest that the TRAF family members play critical roles in the MAVS-mediated signaling pathway in response to viral infection.

The FK506-binding proteins (FKBPs) belong to the immunophilin family and have peptidylprolyl *cis*/*trans* isomerase (PPIase) activity [27]. Currently, 14 FKBPs have been identified in humans. All FKBPs possess at least one conserved FKBP12-like domain, which can be a target region for the immunosuppressive drug FK506. FKBP51, a member of the high-molecular-weight FKBPs, consists of two FKBP12-like domains (FK1 and FK2) and three tetratricopeptide motifs (TPRs) [28], [29]. The FK1 domain is responsible for the PPIase activity of FKBP51 and its binding activity to FK506. In contrast, the TPR motifs are required for its binding to heat shock protein 90 (HSP90). It has been reported that FKBP51 negatively modifies the transcriptional activity of steroid hormone receptors through its interaction with HSP90 by competing with the positive regulatory complex consisting of HSP90 and FKBP52 for the steroid hormone receptor [30]. Moreover, the binding of HSP90 to FKBP51 appears to be critical for the subcellular localization of FKBP51 in the mitochondria [31].

In addition to its function as a co-chaperon of HSP90, FKBP51 acts as a scaffolding protein in the regulation of the activity of protein kinase B (AKT) [29]. FKBP51 inhibits AKT phosphorylation by recruiting PH domain leucine-rich repeat protein phosphatase, thereby reducing AKT activity. Consequently, this mechanism appears to increase the chemosensitivity of tumors. A previous study also revealed that FKBP51 interacts with several serine threonine kinases, including IKKα, TGFβ-activating kinase 1 (TAK1), and MEK kinase 1 (MEKK1), which regulate the activation of NF-κB [32]. The depletion of FKBP51 resulted in the reduction of NF-κB activation induced by TNFα stimulation [32]. FKBP51 is also reported to promote the NF-κB activation induced by ionizing radiation in melanoma [33]. These data suggest that FKBP51 is a modulator of NF-κB activation signaling.

In this study, we identified FKBP51 as a TRAF3- and TRAF6-binding protein. FKBP51 accumulates in the mitochondria and facilitates the expression of type I IFN induced by dsRNA and Newcastle disease virus (NDV). Thus, our data suggest a novel role for FKBP51 in modulating the innate immune responses to viral infection.

## Materials and Methods

### Ethics Statement

All experiments using mice were approved by Committee for Animal Experiments of the Institute of Medical Science, University of Tokyo (approved number H13-26) and were handled in accordance with the Guidelines for Animal Experiments of the Institute of Medical Science, University of Tokyo.

### Cell Cultures, Viruses, Plasmids, and Antibodies

MEF cells and HEK293T cells were described previously [22] and cultured in DMEM supplemented with 10% FBS under 5% CO_2_ at 37°C. L929 cell were described previously [5], [22]. NDV was kindly provided by Dr. T. Abe and Dr. Y. Matsuura. The plasmids have been described previously. Anti-FKBP51 antibody (F14, sc-11518), anti-RelA (C-20, sc-372G), anti-Parp1 (H-300, sc-25780), anti-JNK1 (C-17, sc474), anti-JNK2 (N-18, sc-827), anti-p38a (C-20, sc-535) and anti-Tom20 antibody (FL-145, sc-11415) were obtained from Santa Cruz Biotechnology (Santa Cruz, CA). Anti-phospho JNK (#9251) and anti-phospho p38 (#9211) were from Cell Signaling Technology (Danvers, MA). Anti-tubulin (DM1A) was purchased from Millipore. FKBP51 MEF cells were isolated from FKBP51-deficient mice [34].

### *In Vitro* Virus (IVV) Selection

Randomly primed reverse transcription and the preparation of cDNA were performed with the SuperScript Double-Stranded cDNA Synthesis Kit (Invitrogen, Carlsbad, CA, USA). Reverse transcription was performed using a mouse fetal thymus gland polyA^+^ mRNA library. A synthetic double-stranded adaptor was ligated specifically to the 5′ end of the cDNA. The adaptor-ligated cDNA library was amplified with PCR. The cDNA library was transcribed and ligated to the PEG Puro spacer [35] with the RiboMAX Large Scale RNA Production System–SP6 (Promega) and m7G(5′)ppp(5′)G RNA Cap Structure Analog (Invitrogen Corp.) [36]. The DNA template of (full-length) TRAF6, used as the bait, was prepared with four-step PCR using Ex Taq DNA polymerase (Takara Bio). Overlap PCR was performed to connect the amplicon of the first PCR to that of the second PCR using the program described above. The final PCR was also performed with the same program. The mRNA template of TRAF6, used as bait, was prepared with the RiboMAX Large Scale RNA Production System-SP6 (Promega) and m7G(5′)ppp(5′)G RNA Cap Structure Analog (Invitrogen) [36]. *In vitro* virus (IVV) selection was performed as previously reported [35], [36]. Briefly, the mRNA templates used as bait and prey were cotranslated in wheat germ extract (Molecuence Corporation) for 1 h at 26°C in 96-well plates using the Qiagen BioRobot 8000 system. After four rounds of selection, the interaction sequence tags (ISTs) obtained by IVV selection were identified by Takara Bio Inc., Otsu, Japan, and Shimadzu Corporation, Genomic Research Center, Kyoto, Japan. A mock experiment was performed without the bait protein as the negative control to eliminate technical false-positive results. The ISTs selected with IVV were verified as previously reported [37]. Briefly, the gene corresponding to each IST was assigned with a BLASTN homology search of the coding sequences in the NCBI mouse RefSeq, using the IVV analysis system developed by Fujitsu Limited (IWAS) [36]. ISTs with an E-value ≤1.0E–5 and a match length ≥30 bp were deemed to be positive matches. Frameshift mutants were excluded from our analysis for the purpose of clarity.

### Immunoprecipitation Assay

HEK293T cells were transiently transfected with Flag-tagged and Myc-tagged expression plasmids. At 48 h after transfection, the cells were lysed with TNE buffer (50 mM Tris-HCl [pH 7.5], 150 mM NaCl, 1 mM EDTA, 1% NP-40) supplemented with cOmplete, EDTA-free Protease Inhibitor Cocktail Tablets (Roche) and incubated with anti-Flag antibody (Sigma-Aldrich) at 4°C for 1 h, followed by incubation with Protein G Sepharose 4 FF (GE Healthcare) for 1 h. The immunoprecipitates were washed three times with TNE buffer with protease inhibitors, boiled in SDS sample buffer (65 mM Tris-HCl, 3% SDS, 5% 2-mercaptoethanol, 10% glycerol) for 10 min, and subjected to immunoblotting analysis.

### Silencing of FKBP51 by Small Interfering RNA (siRNA)-mediated Knockdown

MEF cells or L929 cells were transfected with FKBP51-specific Stealth RNA (Invitrogen) using RNAi MAX (Invitrogen). At 48 h after the siRNA treatment, the cells were stimulated with the lipofection of polyriboinosinic polyribocytidylic acids (poly I:C; 10 µg/ml) (Invivogen, San Diego, CA) or NDV infection. The FKBP51-specific Stealth RNA sequences were: fwd: 5′-AACGCCUGAAUCUUC AAAUAAAUCC-3′; rev: 5′-GGAUUUAUUUGAAGAUUCAGGCGUU-3′. Stealth RNA Interference Negative Control Low GC Duplex 3 (Invitrogen) was used as the control.

### Immunoblotting

L929 cells were transfected with poly I:C (10 µg/ml) for the indicated periods with Lipofectamine 2000 reagent (Invitrogen). The cells were lysed in SDS sample buffer for 10 min. The samples were separated on a denaturing polyacrylamide gel containing SDS and then transferred to a polyvinylidene difluoride membrane (Immobilon-P, Millipore). After incubation with TBST (10 mM Tris-HCl, 150 mM NaCl, 0.1% Tween 20) containing 5% nonfat dry milk for 1 h, the membrane was incubated overnight at 4°C with antibodies in TBST containing 5% nonfat dry milk or 5% BSA. The membrane was washed three times with TBST and then incubated with secondary antibody conjugated with horseradish peroxidase. After the membrane was washed three times with TBST, the antibody-specific bands were visualized with the ECL system (GE Healthcare) and the membrane was exposed to Hyperfilm ECS (GE Healthcare) or detected with the Chemi Doc XRS(+) system (Bio-Rad).

### Preparation of Nuclear Fraction

L929 cells were transfected with poly I:C (10 µg/ml) for the indicated periods with Lipofectamine 2000 reagent (Invitrogen). Nuclear extract was prepared according to previously described manner [22]. Cells were suspended in hypotonic buffer containing 10 mM HEPES (pH 7.9), 1.5 mM MgCl2, 10 mM KCl, 0.5 mM dithiothreitol (DTT), and protease inhibitor cocktail (Roche). The cell suspension was incubated on ice for 20 min and was subsequently disrupted with pipetting. The supernatant was removed, and the pelleted nuclei were incubated with nuclear extraction buffer containing 20 mM HEPES (pH 7.9), 1.5 mM MgCl2, 420 mM NaCl, 0.2 mM EDTA, 0.5 mM DTT, protease inhibitor cocktail and 25% glycerol. The suspension was maintained on ice for 20 min, and the nuclear extract was obtained from the supernatant.

### Infection with Virus and Transfection with dsRNA

For viral infection, the cells were incubated with NDV at a multiplicity of infection (MOI)  = 5 for 1 h in DMEM without FBS, which was then replaced with DMEM containing 10% FBS. Excess virus was washed off 1 h after infection. The MEF cells were infected with NDV for 8 h or transfected with 10 µg/ml poly I:C (Invivogen, San Diego, CA) or 10 µg/ml B-DNA IFN stimulatory DNA (ISD) [33] for 4 h using Lipofectamine 2000 (Invitrogen).

### Real-time PCR Analysis

Total RNA was isolated from cells using TRIzol Reagent (Life Technologies). cDNA was synthesized using PrimeScript II (Takara Bio). A quantitative real-time PCR analysis was performed with the Applied Biosystems 7300 Real Time PCR System and Thunderbird SYBR qPCR Mix (Toyobo, Osaka, Japan). The expression level of *Gapdh* or *Actb* was used to normalize the data. The primers used were Actinb: 5′-GGCTGTATTCCCCTCCATCG-3′ and 5′-CCAGTTGGTAACAATGCCATGT-3′; Gapdh: 5′-ACCATGTAGTTGAGGTCAATGAAGG-3′ and 5′-GGTGAAGGTCGGTGTGAACG-3′; IFNβ: 5′-AGCTCCAAGAAAGGACGAACA-3′ and 5′-GCCCTGTAGGTGAGGTTGATC-3′; Isg15: 5′-GACTAACTCCATGACGGTG-3′ and 5′-AACTGGTCTTCGTGACTTG-3′.

### Confocal Microscopy

MEF cells were seeded on cover glasses. The cells were transfected with poly I:C (10 µg/ml) using Lipofectamine 2000 (Invitrogen). Four hours after transfection, the cells were fixed with ice-cold acetone for 5 min. After incubation with blocking buffer (PBS containing 3% BSA), the cells were incubated overnight at 4°C with anti-FKBP51 antibody (F14) and anti-Tom20 antibody in PBS containing 3% BSA, followed by Alexa-Fluor-labeled donkey anti-goat antibody or donkey anti-rabbit antibody at room temperature for 1 h. In some experiments, the nuclei were stained with a combination of propidium iodide (1 µg/ml) and RNase A. The stained cells were mounted on slide glasses with mounting medium (80% glycerol with 1% n-propyl gallate). Confocal color images were obtained with an Olympus FV1000D microscope.

### Luciferase Assay

HEK293T cells were transfected with IFN-stimulated response element (ISRE)–luciferase, effector plasmid and a β-actin-promoter-driven β-galactosidase (β-gal)-coding plasmid as the internal control, using the calcium phosphate method. The total amounts of transfected plasmids were made identical by adding each control vector. At 48 h after transfection, the cells were lysed and subjected to a PicaGene luciferase assay (Toyo Ink, Tokyo, Japan). Relative luminescence units were measured with a luminometer (Lumat LB9507, EG&G Berthhold). Transfection efficiency was normalized by measuring the β-gal activity using O-nitrophenyl galactoside as the substrate.

## Results

### Identification of FKBP51 as a Novel TRAF6-binding Protein with IVV Selection

**Figure 1:**
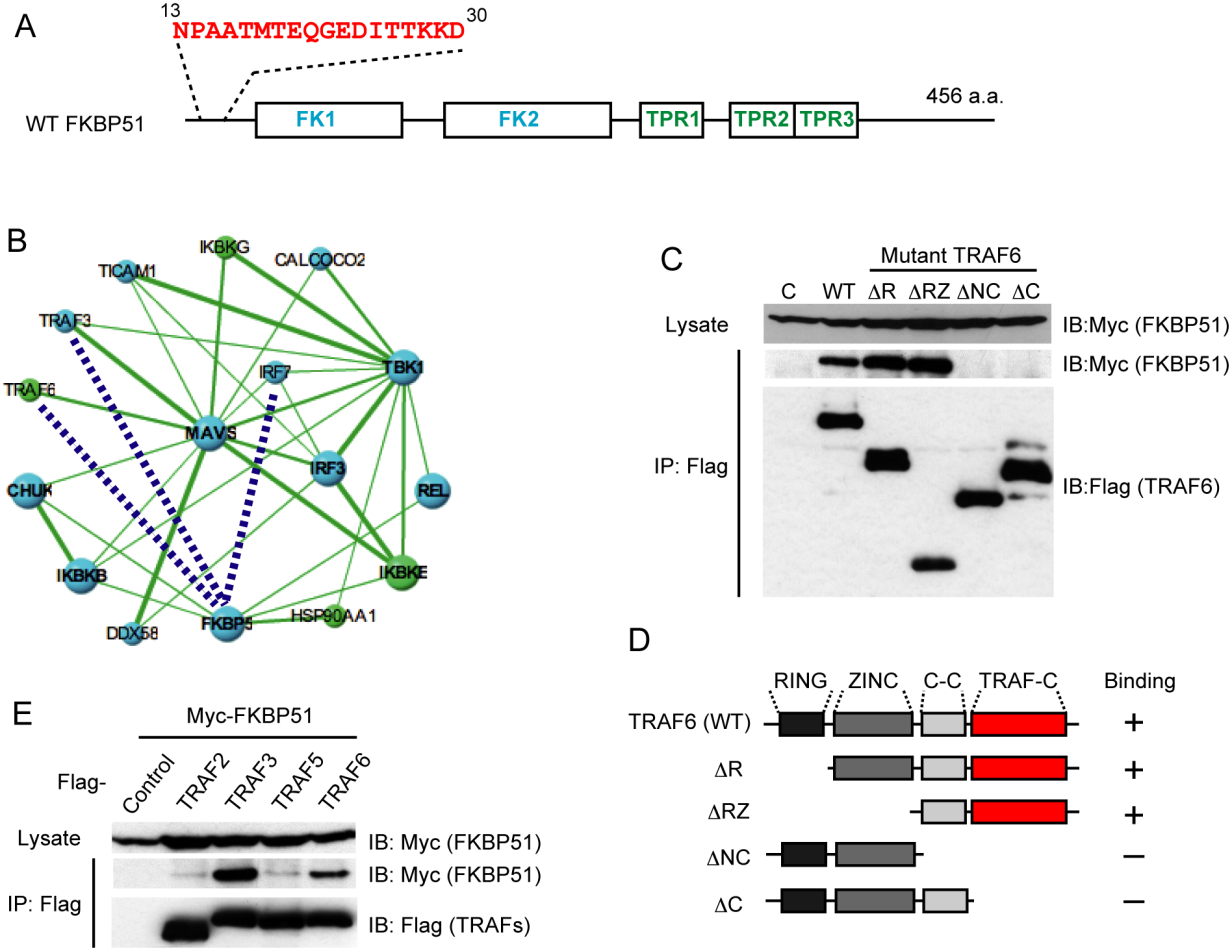
FKBP51 is a novel TRAF6-binding protein. (A) The schematic structure of mouse FKBP51. FK1 and FK2 indicate FKBP12-like domains. TPR1, TPR2, and TPR3 indicate tetratricopeptide motifs. An amino acid sequence showing a peptide determined with IVV selection to be a TRAF6-binding peptide. (B) Protein–protein interaction network in MAVS-mediated type I IFN induction obtained from the PPI database (http://genomenetwork.nig.ac.jp/index_e.html). The three dotted lines show the interactions newly identified in this study. The red dotted line indicates the interaction identified with IVV selection. (C) Coimmunoprecipitation of FKBP51 with TRAF6 and its mutants (ΔR, ΔRZ, ΔNC, and ΔC). The TRAF6 mutants expressed in the cells are indicated on the top of the panels. WT and C indicate full-length TRAF6 and Flag-tagged expression vector, respectively. The upper panel shows the western blotting of immunoprecipitates using anti-Myc antibody to detect Myc-tagged FKBP51. Middle panels show the western blotting of immunoprecipitates using anti-Myc antibody to detect Flag-tagged TRAF6 and mutants. The lower panel shows the western blotting of total cell lysates using anti-Myc antibody. One representative experiment of three is shown. (D) Schematic structures of TRAF6 and its deletion mutants used in this study. “RING” indicates the RING-finger domain. “ZINC” indicates the region of six zinc-finger domains. “C-C” indicates the coiled-coil domain. “TRAF-C” indicates the TRAF domain conserved in all TRAF family members. The Flag tag (abbreviated in this figure) was connected to the N-terminal of the WT protein and mutants. The binding ability of each protein to FKBP51, as determined in Figure 1C, is indicated on the right of each structure. “+” indicates positive for binding, and “–” indicates negative for binding. (E) FKBP51 preferentially binds TRAF3 and TRAF6. Flag-tagged TRAF proteins and Myc-tagged FKBP51 were expressed in HEK293T cells and immunoprecipitated with an anti-Flag antibody. The TRAF proteins expressed in the cells are indicated on the top of the panels. The upper panel shows the western blotting of samples immunoprecipitated with anti-Myc to detect FKBP51. The middle panel shows the western blotting of samples immunoprecipitated with anti-Flag antibody. The lower panel shows the western blotting of the cell lysate with anti-Myc antibody. One representative experiment of three is shown.

We previously demonstrated that TRAF6 promotes the induction of type I IFN and proinflammatory cytokines upon sensing cytosolic dsRNA or DNA [22]. Although TRAF6 is reported to bind to MAVS and IRF7 [38], the molecular mechanism by which TRAF6 activates the downstream signaling to regulate type I IFN expression remains unclear. We first looked for previously unidentified TRAF6-binding proteins that potentially regulate type I IFN expression. We used IVV selection of the interacting peptides [36], which included several steps: *in vitro* transcription of the bait and prey cDNAs; cell-free cotranslation, IVV selection, and amplification of the selected IVVs by RT-PCR. Multiple rounds of this selection procedure can be used to detect relatively weak interactions. We screened a cDNA library from the murine thymus because the expression of IFNβ in the thymus is reported to be constitutively high [38]. IVV selection identified several TRAF6-binding peptides. The candidate TRAF6-binding proteins containing these peptides are summarized in Table 1. Of these new candidate TRAF6-binding proteins, we chose FKBP51 (Fig. 1A) for further analysis because FKBP51 is reported to bind to IKKε[32] and HSP90 [30], which regulate the activation of IRF3 [39]. However, the role of FKBP51 in the type I IFN expression induced by cytosolic nucleic acids has not been determined. Interestingly, an *in silico* analysis using a protein–protein interaction (PPI) network database (http://genomenetwork.nig.ac.jp/index_e.html) implied that FKBP51 (FKBP5) is a component of a PPI network composed of a set of proteins that regulate the induction of type I IFN (Fig. 1B).

**Table 1:** List of genes identified as TRAF6-binding protein candidates.

Gene symbol	Gene name
Ctcf	CCCTC-binding factor
Eml2	echinoderm microtubule associated protein like 2
Fkbp5	FK506 binding protein 5
Gltscr2	glioma tumor suppressor candidate region gene 2
Hdac2	histone deacetylase 2
Jun	Jun oncogene
Jund	Jun proto-oncogene related gene d
Ngfrap1	nerve growth factor receptor (TNFRSF16) associated protein 1
Phpt1	phosphohistidine phosphatase 1
Parp1	poly (ADP-ribose) polymerase family, member 1
Psmb1	proteasome (prosome, macropain) subunit, beta type 1
Rbm39	RNA binding motif protein 39
Sfpq	Sfpq splicing factor proline/glutamine rich (polypyrimidine tract binding protein associated)
Supt16 h	suppressor of Ty 16 homolog (S. cerevisiae)
Top1	topoisomerase (DNA) I
Tloc1	translocation protein 1
Trim26	tripartite motif protein 26
Whdc1	WAS protein homology region 2 domain containing 1
Zmiz1	zinc finger, MIZ-type containing 1

The TRAF6-binding sequence of FKBP51 determined by IVV selection is located in the N-terminal flanking region of the FK1 domain of FKBP51 (Fig. 1A). Interestingly, neither the region determined with IVV selection nor the whole amino acid sequence of FKBP51 contains the consensus amino acid sequence for TRAF6 binding (PxExxAc/Ar) [40] or that for binding to other TRAF family members (PxQxT/S) [41], suggesting that FKBP51 binds to TRAF6 in a unique way.

### Binding of FKBP51 to TRAF3 and TRAF6 in Cells

We next verified that the TRAF6–FKBP5 interaction determined in the *in vitro* system also occurs in living cells. Flag-tagged TRAF6 and Myc-tagged FKBP51 were coexpressed in HEK293T cells. A coimmunoprecipitation assay demonstrated that FKBP51 interacts with TRAF6 in living cells (Fig. 1C; two left lanes). To gain some insight into the function of FKBP51 in TRAF6-mediated signaling, we identified the domains in TRAF6 responsible for its binding to FKBP51. TRAF6 is divided into several structural domains: a RING-finger domain, a domain containing six consecutive zinc fingers, a coiled-coil domain, and a TRAF-C (MATH) domain (Fig. 1D) [42]. The RING-finger domain confers E3 ligase activity on TRAF6 and the RING and zinc fingers are reported to be critical for the activation of downstream signaling [43]. In contrast, the coiled-coil and TRAF-C domains facilitate the oligomerization of TRAF6 and its binding to upstream receptors or adaptor proteins [42]. Several deletion mutants of TRAF6 (ΔR, ΔRZ, ΔNC, and ΔC in Fig. 1D) were coexpressed with FKBP51 in HEK293T cells. Coimmunoprecipitation experiments demonstrated that the deletion of the RING-finger and zinc-finger domains (ΔR and ΔRZ) did not impair its interaction with FKBP51 (Fig. 1D). In contrast, mutants lacking both the coiled-coil and TRAF-C domains (ΔNC) or lacking only the TRAF-C domain (ΔC) failed to interact with FKBP51 (Fig. 1D). Therefore, FKBP51 probably binds to the TRAF-C domain, implying that FKBP51 functions as a regulator or adaptor of the TRAF6-mediated signaling pathway.

The TRAF-C domain is conserved among TRAF family proteins [44]. Moreover, in addition to TRAF6, TRAF2, 3, and 5 are reported to regulate MAVS-mediated signaling [12], [21], [25], [26]. Therefore, we next examined whether these TRAF family proteins bind to FKBP51. Coimmunoprecipitation experiments suggested that TRAF3 binds to FKBP5 more efficiently than TRAF2 or TRAF5 (Fig. 1E). Therefore, of the TRAF family proteins, FKBP51 might selectively interact with TRAF3 and TRAF6.

### FKBP51 Facilitates the Expression of Type I IFN Induced by Virus Infection and Cytosolic dsRNA

**Figure 2:**
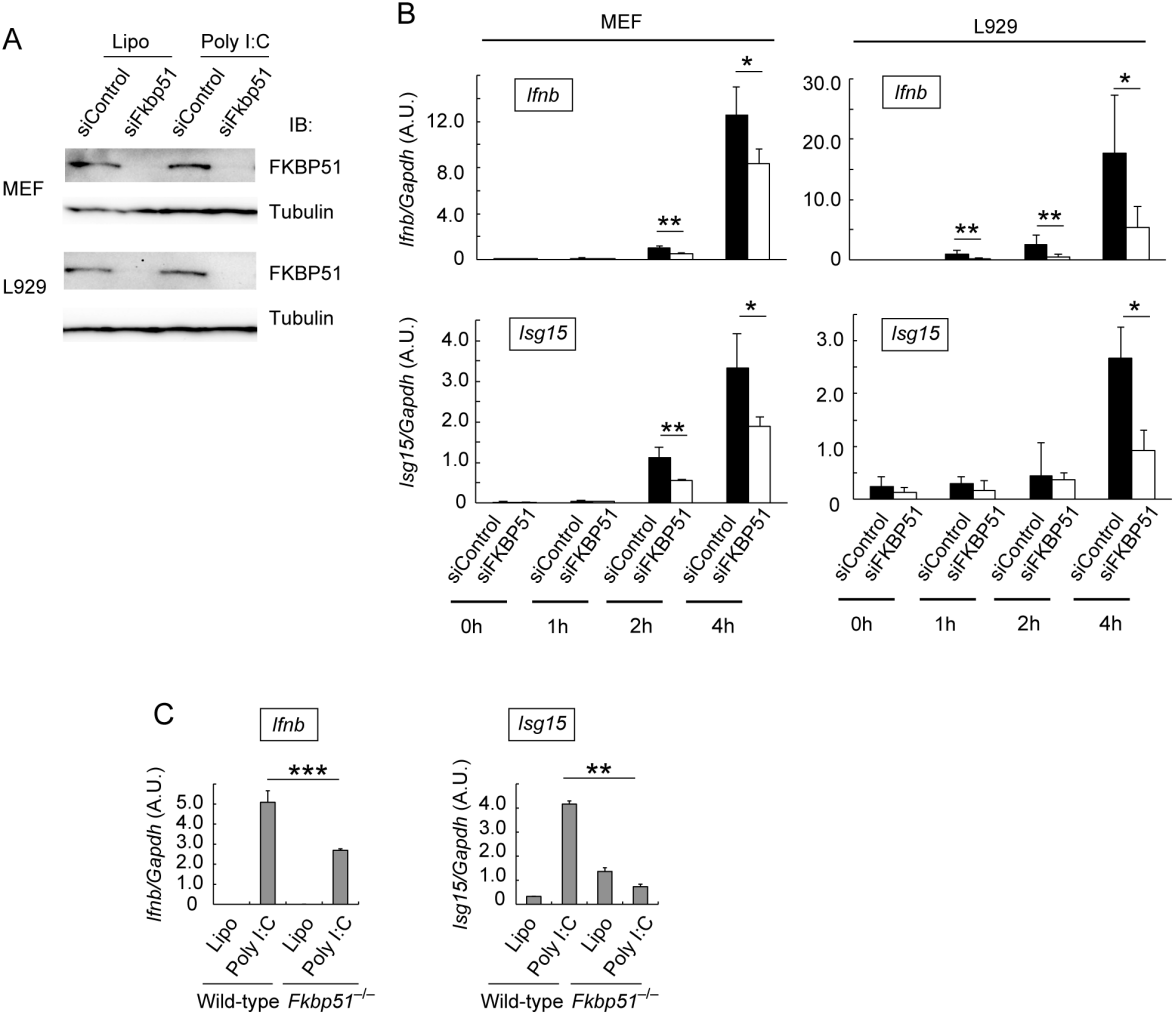
FKBP51 facilitates the cytosolic poly I:C-dependent expression of type I IFN. (A) Knockdown of FKBP51 in MEF cells and L929 cells. Total cell lysates from FKBP51-knockdown cells (siFKBP51) and control cells (siControl) stimulated by lipofection with poly I:C or control lipofection were analyzed with western blotting using anti-FKBP51 antibody (upper panel) or anti-tubulin antibody (lower panel) as the loading control. (B) Reduction in the expression of IFNβ and ISG15 in FKBP51-knockdown MEF cells and L929 cells stimulated by lipofection with poly I:C. FKBP51-knockdown or control MEF and L929 cells were unstimulated or stimulated by lipofection with poly I:C for 1, 2 and 4 h. The expression levels of IFNβ (*Ifnb*) and ISG15 (*Isg15*) were evaluated with a qPCR analysis and normalized to the level of GAPDH mRNA. Data are the means ± SD of triplicate determinations. *P<0.05 and **P<0.01; Student’s *t* test with a two-tailed distribution and two-sample equivalent variance parameters. (C) Reduction in the expression of IFNβ in FKBP51-deficient (*Fkbp51* ^−/−^) MEF cells stimulated with dsRNA. FKBP51-deficient and control MEF cells were transfected with poly I:C by lipofection. The expression of IFNβ and ISG15 was evaluated with a qPCR analysis. Data are the means ± SD of triplicate determinations and are representative of three independent experiments. ***P<0.001 and **P<0.01; Student’s *t* test with a two-tailed distribution and two-sample equivalent variance parameters.

We next examined whether FKBP51 is involved in the induction of type I IFN by sensing cytosolic nucleic acids. We took advantage of the siRNA-mediated knockdown of FKBP51 to investigate the roles of FKBP51 in response to cytosolic dsRNA. A western blotting analysis confirmed that FKBP51-directed siRNA efficiently reduced the expression of FKBP51 in MEF cells and the L929 fibroblast cell line (Fig. 2A). Lipofection of synthetic dsRNA (poly I:C) is known to elicit the expression of IFNβ by activating the MAVS complex through RLR-dependent sensing. Quantitative RT-PCR analysis (qPCR) indicated that the poly I:C dependent expression of IFNβ and ISG15, which is a type-I-IFN-stimulatory protein, was significantly diminished by silencing FKBP51 expression in MEF and L929 cells (Fig. 2B). Reductions in IFNβ expression by the FKBP51-depletion were more significant in early phase of poly I:C stimulation. This implies that silencing FKBP51 expression might cause a delay of IFNβ expression induced by dsRNA sensing. In addition to siRNA-mediated knockdown, the expression of IFNβ and ISG15 induced by poly I:C lipofection was significantly reduced in MEFs derived from FKBP51-deficient mice (Fig. 2C). Overall, we concluded that FKBP51 facilitates the expression of type I IFN induced by cytosolic dsRNA.

**Figure 3:**
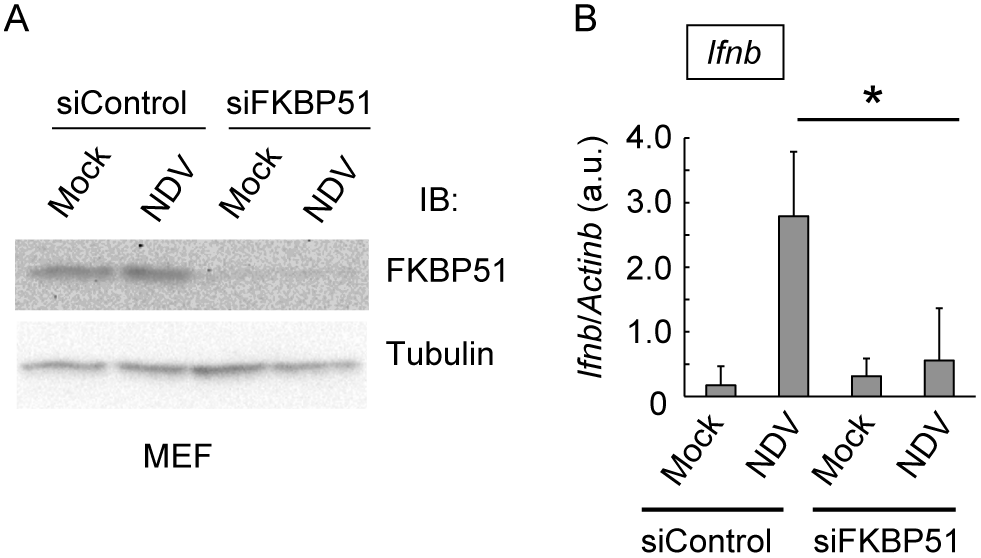
FKBP51 enhances NDV-dependent expression of type I IFN. (A) Knockdown of FKBP51 in MEF cells. Total cell lysates from FKBP51-knockdown cells (siFKBP51) and control knockdown cells (siControl) infected with NDV or without infection were analyzed by western blotting using anti-FKBP51 antibody (upper panel) and anti-tubulin antibody (lower panel) as the loading control. One representative experiment of three is shown. (B) FKBP51-knockdown and control MEF cells were infected with NDV (MOI = 10). At 8 h after infection, the expression of IFNβ was evaluated by qPCR and normalized to the expression of β-actin mRNA. Data are the means ± SD of triplicate determinations and are representative of three independent experiments. *P<0.05; Student’s *t* test with a two-tailed distribution and two-sample equivalent variance parameters.

We next examined whether FKBP51 promotes the expression of type I IFN triggered by RNA viral infection. MEFs were infected with NDV, which induces type I IFN expression through MAVS complex signaling in an RIG-I-dependent manner. qPCR analysis indicated that the NDV-induced expression of IFNβ was significantly reduced in FKBP51-depleted MEF cells (Fig. 3). These data suggest that FKBP51 facilitates the expression of type I IFN induced by viral infection.

### FKBP51 Enhances Activations of Signaling Pathway Induced by Cytosolic dsRNA

**Figure 4:**
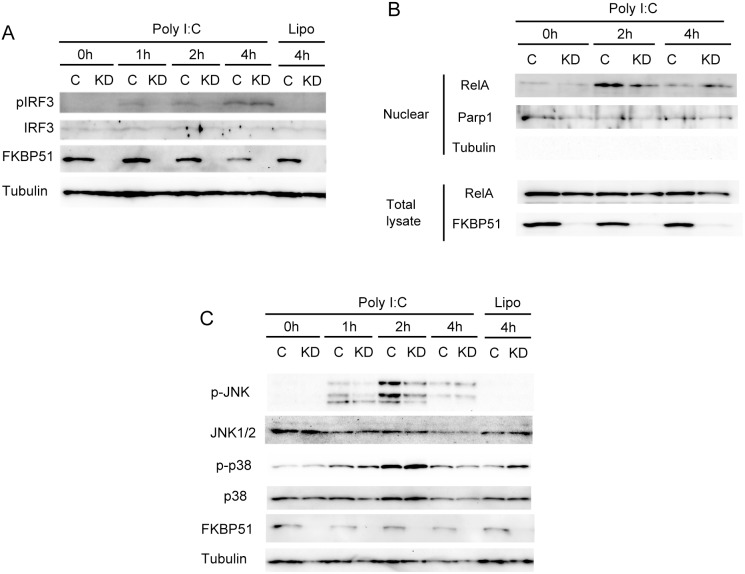
FKBP51 enhances the cytosolic poly I:C-dependent activations of IRF3, RelA, and JNK. (A) Immunoblotting analysis of total cell lysates from FKBP51-knockdown or control MEF cells stimulated by lipofection with or without poly I:C. Total cell lysates were recovered at various time points after stimulation and analyzed by western blotting using anti-phospho IRF3, anti-IRF3, anti-FKBP51 and anti-tubulin antibodies. (B) Immunoblotting analysis of total cell lysates (whole) and nuclear extracts (Nuclear) from FKBP51-knockdown or control MEF cells stimulated by lipofectamin with poly I:C. Total cell lysates and nuclear extracts were recovered at various time points after poly I:C stimulation and analyzed by anti-RelA. Anti-Parp1 was used for loading control of nuclear fraction. Anti-tubulin was used to monitor contamination of cytosolic fraction. (C) Immunoblotting analysis of total cell lysates from FKBP51-knockdown or control MEF cells stimulated by Lipofection with or without poly I:C. Total cell lysates were recovered at various time points after poly I:C stimulation and analyzed by western blotting using anti-phospho p38, anti-p38, anti-phospho JNK, anti-JNK1 and 2, anti-FKBP51, and anti-tubulin antibodies.

The binding of dsRNAs to RLRs triggers the formation of the MAVS complex. The formation of the MAVS complex is known to lead to the phosphorylation of IRF3, mediated by the atypical IKKs, thereby initiating the expression of type I IFN. We investigated whether FKBP51 modulates the phosphorylation of IRF3 induced by dsRNA. A western blotting analysis showed that poly I:C stimulation triggered the phosphorylation of IRF3 1 h after stimulation. Interestingly, FKBP51 depletion delayed the initiation of IRF3 phosphorylation by 4 h after poly I:C stimulation (Fig. 4A). This suggests that FKBP51 expedites the phosphorylation of IRF3 induced by dsRNA sensing.

Besides IRF3 activation, the RLR-mediated signaling induces NF-κB activation that promotes expressions of type I IFNs [14]. Moreover, previous studies showed that FKBP51 enhances the NF-κB activation [32], [33]. We therefore investigated whether FKBP51 is involved in RLR-dependent activation of NF-κB. FKBP51 depletion results in a reduction of RelA nuclear translocation induced by the 2 h poly I:C stimulation (Fig. 4B). Interestingly, such reduction was not observed 4 h after the poly I:C stimulation (Fig. 4B). Thus, data suggest that FKBP51 enhances early activation of NF-κB triggered by the RLR-mediated signaling.

Finally, we address whether FKBP51 is involved in MAPK signaling. Transfection of poly I:C leads to the activations of p38 MAPK and JNK [23], [24]. A western blotting analysis showed that depletion of FKBP51 causes a reduction of JNK phosphorylation induced by the poly I:C stimulation (Fig. 4C). This inhibition occurred by 2 h but was not detected 4 h after the poly I:C stimulation. On the other hand, the poly I:C-dependent phosphorylation of p38 MAPK was not practically influenced by the absence of FKBP51 (Fig. 4C). Overall, these data suggest that FKBP51 enhances the RLR-mediated activations of IRF3, NF-κB and JNK in early phase of poly I:C stimulation. These findings are consistent with the idea that FKBP51 expedites IFNβ induction by sensing dsRNAs.

### FKBP51 is Preferentially Localized in the Mitochondria after dsRNA Sensing

**Figure 5:**
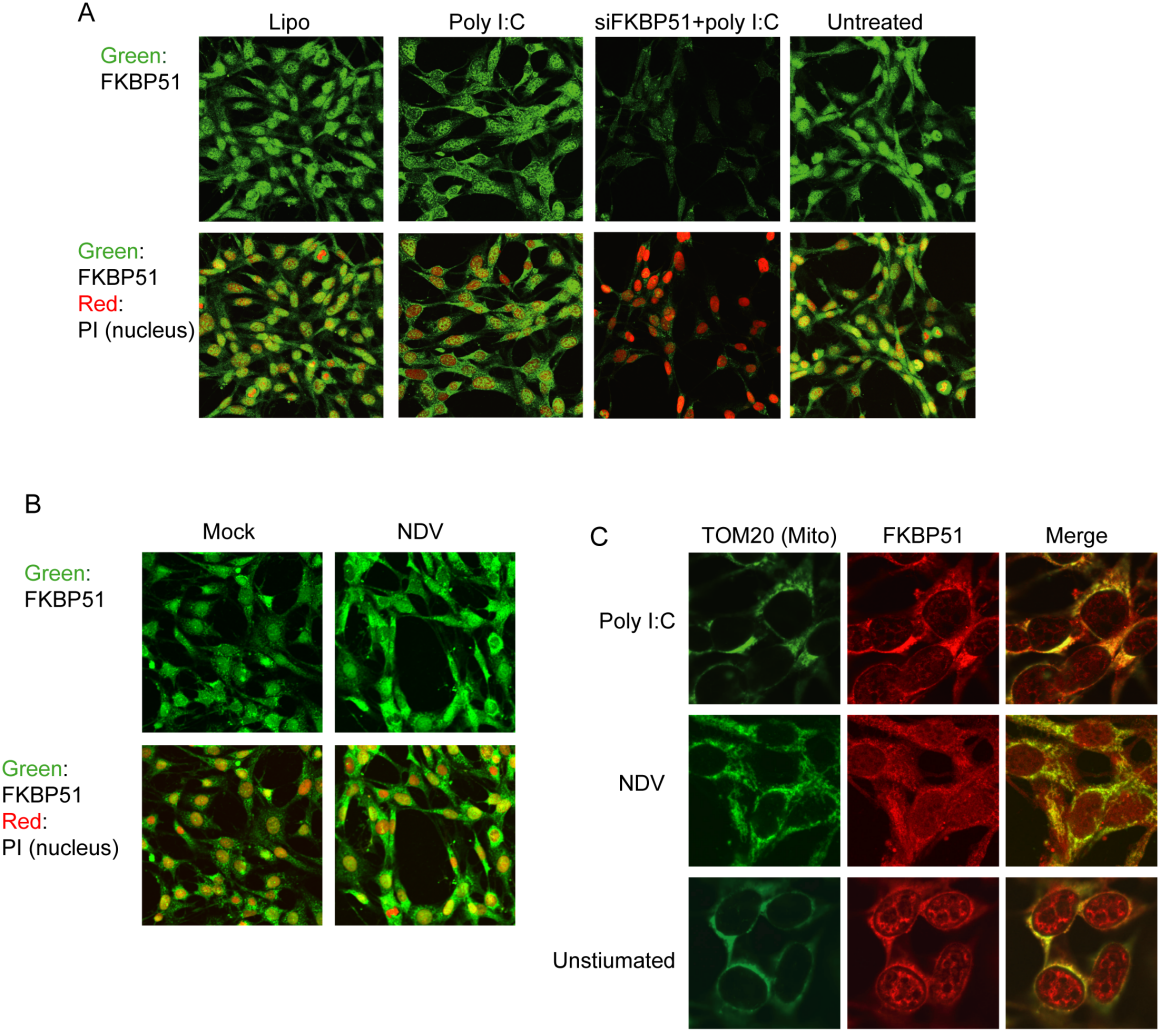
FKBP51 preferentially accumulates in the mitochondria after cytosolic dsRNA stimulation. (A) Cytoplasmic accumulation of FKBP51 in MEF cells after stimulation with cytosolic dsRNA. MEF cells were stimulated by Lipofectamine with or without poly I:C. Endogenous FKBP51 was detected with an anti-FKBP51 antibody. The nuclei were visualized by staining with propidium iodide. One representative experiment of three is shown. (B) Cytoplasmic accumulation of FKBP51 in MEF cells after NDV infection. Endogenous FKBP51 was detected with an anti-FKBP51 antibody. The nuclei were visualized by staining with propidium iodide. One representative experiment of three is shown. (C) Subcellular localization of FKBP51 in mitochondria. MEF cells were stimulated by lipofectamine with poly I:C or NDV infection. Endogenous FKBP51 was detected with an anti-FKBP51 antibody. The mitochondria were visualized by immunostaining with anti-TOM20 antibody. One representative experiment of three is shown.

Recent studies have shown that the mitochondria participate in a wide range of innate immune responses [45]. For instance, the multiprotein MAVS complex is formed on the outer mitochondrial membrane after dsRNA sensing. Interestingly, FKBP51 was recently reported to localize to both the nucleus and mitochondria of cells [31]. It has also been shown that the subcellular localization of FKBP51 is dynamically altered by cellular conditions [31]. Therefore, FKBP51 appears to shuttle between the nuclei and mitochondria. Consistent with previous findings, FKBP51 was detected in both the nucleus and cytoplasm when MEF cells were immunostained for endogenous FKBP51 (Fig. 5A; Untreated). Interestingly, the stimulation of MEF cells with poly I:C caused a reduction in the nuclear staining of FKBP51 and an increase in cytoplasmic FKBP51 staining (Fig. 5A; middle left) compared with the lipofection control (Fig. 5A; left), suggesting that a proportion of FKBP51 is translocated from the nucleus to the cytoplasm by dsRNA stimulation. Consistent with this, NDV infection also caused the preferential accumulation of FKBP51 in the cytoplasm (Fig. 5B). The immunostaining for FKBP51 in the cytoplasm was coincident with immunostaining for the mitochondrial protein Tom20 in cells treated with poly I:C lipofection (Fig. 5C). These data suggest that FKBP51 preferentially accumulates in the mitochondria after dsRNA sensing. Interestingly, the mitochondrial accumulation of FKBP51 occurred in the absence of both TRAF3 and TRAF6 (Figure S1). Thus, the interaction between FKBP51 and TRAF3 and 6 may not control the localization of FKBP51 but rather may modulate activations of these TRAF proteins.

### FKBP51 might be a Scaffold Protein Regulating MAVS-mediated Signaling

**Figure 6:**
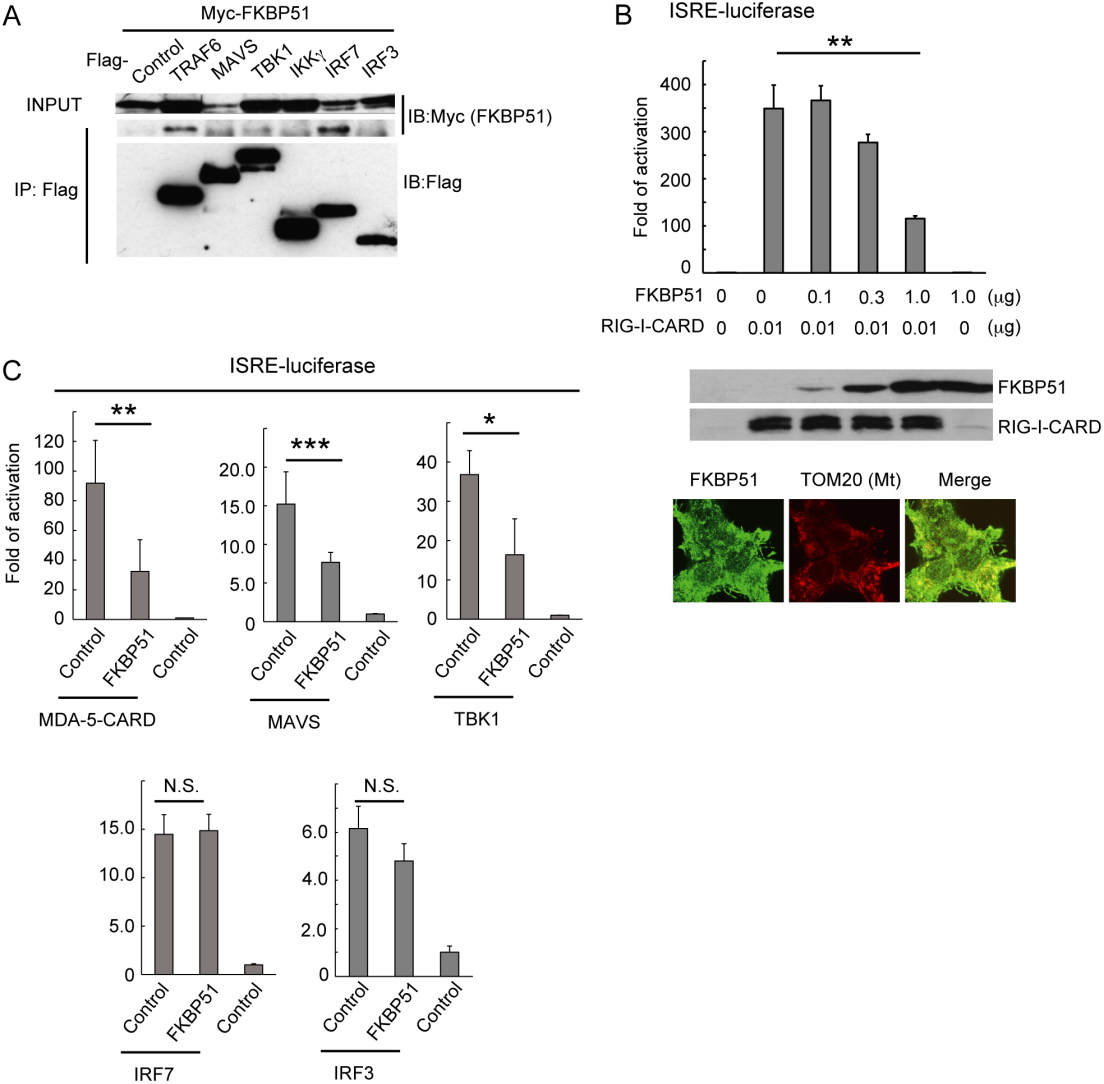
FKBP51 is a possible scaffolding protein in RIG-I-dependent signaling. (A) Binding of FKBP51 to IRF7 in HEK293T cells. Combinations of Myc-tagged FKBP51 and Flag-tagged TRAF6, MAVS, TBK1, NEMO, IRF7, IRF3, or control were transiently expressed in HEK293T cells. The transfected genes are indicated on the top of the panels. IP and INPUT panels indicate the western blotting analysis of the immunoprecipitated samples and the cell lysates used for immunoprecipitation, respectively. The antibodies used for western blotting are shown on the right of the panels. One representative experiment of two independent experiments is shown. (B) Luciferase activity of HEK293T cells transfected with a combination of plasmids encoding Myc-tagged FKBP51 or control Myc, the Flag-tagged CARD domain of RIG-I (RIG-I-CARD) or control Flag, ISRE-driven luciferase, and β-actin-promoter-driven β-gal. The amount of transfected plasmid encoding FKBP51 or RIG-I-CARD is indicated below the graph. Relative luminescence units (RLU) were normalized to the activity of β-gal. The fold activation was determined as the normalized RLU for each sample relative to those of the sample transfected with the control vector, which indicated the basal activation. Data are the means ± SD of triplicate determinations and are representative of three independent experiments. **P<0.01; Student’s *t* test with a two-tailed distribution and two-sample equivalent variance parameters. Western blotting analysis of transfected samples using anti-Myc antibody or anti-Flag antibody. Subcellular localization of overexpressed FKBP51 is shown in the bottom panels. (C) Luciferase activity of HEK293T cells transfected with combinations of two plasmids encoding FKBP51 and the CARD domain of MDA-5 (MDA-5-CARD), TBK1, MAVS, IRF7, IRF3 or its control vector. The combinations of the transfected plasmids are indicated. Luciferase activity is shown as the fold induction relative to the basal activation, as in Fig. 6B. Data are the means ± SD of triplicate determinations and are representative of three independent experiments. **P<0.01, *P<0.05; Student’s *t* test with a two-tailed distribution and two-sample equivalent variance parameters.

A previous study demonstrated that FKBP51 binds to HSP90 through its C-terminal TPR motifs. HSP90 is reported to interact with TBK1 and IRF3 to promote the expression of type I IFN. In addition to the TRAF proteins and HSP90, we also investigated the interactions of FKBP51 with other MAVS-signaling-related proteins (Fig. 6A). Coimmunoprecipitation experiments suggested that FKBP51 interacts with IRF7 (Fig. 6A), a transcription factor that acts downstream from MAVS-mediated signaling. Together with the binding of FKBP51 to TRAF3 and TRAF6, these data suggest that FKBP51 interacts with a wide variety of proteins to regulate signaling downstream from cytosolic nucleic acid sensors. Therefore, we speculate that FKBP51 acts as a scaffolding protein to enhance the protein–protein interactions that control type I IFN expression. Several studies have shown that the stoichiometric expression of a scaffold protein and its binding proteins is essential to enhance the output signals along a scaffold-associated pathway [46], [47]. Therefore, it was previously proposed that the inhibition of output signaling by the overexpression of a candidate protein may be one criterion defining a scaffold protein [46]. We tested whether the overexpression of FKBP51 inhibits RIG-I-mediated signaling. The expression of RIG-I lacking the C-terminal domain (RIG-I-CARD) is known to activate MAVS-mediated signaling [5]. We used a luciferase reporter gene linked to ISRE to monitor the activation of MAVS signaling. The results suggested that the strong expression of FKBP51 inhibited the ISRE activation induced by the expression of RIG-I-CARD and MAVS in HEK293T cells. The subcellular localization of FKBP51 was in cytoplasmic regions and the mitochondria, suggesting the aberrant localization of the overexpressed FKBP51 (Fig. 6B). The overexpression of FKBP51 inhibits the activation of ISRE induced by the expression of TBK1 or the CARD domain of MDA-5 (Fig. 6C). These data are consistent with idea that FKBP51 acts as a scaffold protein in the MAVS-mediated induction of type I IFN. In contrast, FKBP51 overexpression did not interfere with the IRF7- and IRF3-dependent activation of ISRE (Fig. 6C). This is probably because the expression of type I IFN promoted by the overexpression of IRF7 does not require the activation of upstream signaling.

## Discussion

Sensing cytosolic dsRNAs triggers formation of MAVS complex[9]–[12]. Whereas MAVS complex leads to activations of IRF3, IRF7 and NF-κB, its precise mechanism remains elusive. Our data suggest a novel role for FKBP51 in expression of type I IFN induced by dsRNA sensors. FKBP51 binds to TRAF3 and TRAF6, components of MAVS complex[14]. On the other hand, FKBP51 reportedly binds to IKKε [32] and HSP90 [30] that interacts with IRF3 and TBK1[39]. Moreover, we found that FKBP51 binds to IRF7. Thus, FKBP51 might directly and indirectly interact with MAVS complex and its downstream effectors. Therefore, FKBP51 probably functions as a scaffold protein, bound to several proteins such as TRAF3, TRAF6, IRF7, and the HSP90–IRF3–TBK1/IKKε complex. The function of FKBP51 as a scaffold protein may enforce the interaction between MAVS complex and its downstream effectors, thereby promoting rapid induction of IFNβ.

Although the depletion of FKBP51 downregulated the expression of type I IFN induced by the lipofection of nucleic acids or infection with NDV, this reduction was only partial. Consistent with this, the phosphorylation of IRF3 induced by dsRNA was not completely abolished but was only delayed by the depletion of FKBP51. One possible explanation of this partial defect is that other FKBP proteins have functions similar to that of FKBP51 and partially compensate for the lack of FKBP51. It will be important for a future study to determine whether other FKBP family proteins are involved in MAVS-mediated signaling. Another possibility is that FKBP51 is only required for the early response to cytosolic nucleic acids, and is not essential for the activation of MAVS-mediated signaling. To form the MAVS complex, several signal transducers must move from the cytosol and localize to the surface of the outer mitochondrial membrane. FKBP51 may help these signaling molecules to translocate to the mitochondrial surface. Because this translocation and complex formation might be driven by the simple diffusion of the proteins in the cytoplasm, FKBP51 is not essential for the late stage of MAVS signaling. This idea is consistent with the observation that the phosphorylation of IRF3 is only impaired during the early period of signaling by FKBP51 depletion.

Many receptors and adaptor proteins are known to bind to TRAF3[44] and TRAF6[42], which usually occurs through the interaction of consensus amino acid sequences in the interacting proteins and in the TRAF-C domain. Interestingly, although FKBP51 has no consensus binding motif required to bind the TRAF family proteins, it still interacts with the TRAF-C domain of TRAF6. Importantly, this finding suggests that the binding of FKBP51 does not compete directly with the binding of other TRAF3- and TRAF6-interacting proteins that contain TRAF family consensus motifs. This binding property of FKBP51 may be necessary for its scaffolding function, because it must bind to several types of proteins simultaneously.

Although all TRAF proteins have a similar TRAF-C domain, we found selective binding of FKBP51 with TRAF3 and TRAF6 through TRAF-C domain. Interestingly, however, there seems to be no consensus amino acid sequence distinctive of TRAF-C domains of TRAF3 and TRAF6. Thus, it is likely that FKBP51 recognizes a 3-dimensional structure specific for TRAF-C domains of TRAF3 and TRAF6. Identification of the FKBP51-binding region in the TRAF-C domains might be important future research in order to determine in detail the mode of binding between FKBP51 and TRAF3 or TRAF6.

Overall, our study suggests that FKBP51 is a novel scaffolding protein that enhances the expression of type I IFN induced by cytosolic nucleic acids. Greater understanding of the molecular mechanism associated with this FKBP51-mediated process should contribute to the development of therapies against virus-induced diseases and inflammation.

## Supporting Information

Figure S1 **TRAF3 and TRAF6 are dispensable for mitochondrial accumulation of FKBP51 induced by cytosolic dsRNA.** (A) Knockdown of TRAF3 in MEF cells. Total cell lysates from TRAF3-knockdown MEF cells (left panel; siTRAF3), control MEF cells (left panel; siControl), TRAF3-knockdown TRAF6-deficient MEF cells (right panel; siTRAF3), and control TRAF6-deficient MEF cells (right panel; siControl) were analyzed with western blotting using anti-TRAF3 antibody (upper panel) or anti-tubulin antibody (lower panel) as the loading control. (B) Cytoplasmic accumulation of FKBP51 in MEF cells after stimulation with cytosolic dsRNA. Wild-type MEF cells, TRAF3-knockdown MEF cells, TRAF6-deficient MEF cells, TRAF3-knockdown TRAF6-deficient MEF cells were stimulated by lipofectamine with or without poly I:C. Endogenous FKBP51 was detected with an anti-FKBP51 antibody. (C) TRAF3- knockdown MEF cells (left figure), control MEF cells (left figure), TRAF3-knockdown TRAF6-deficient MEF cells (right figure) and control TRAF6-deficient MEF cells (right figure) were stimulated by lipofectamine with or without poly I:C. The expression levels of IFNβ was evaluated with a qPCR analysis and normalized to the level of GAPDH mRNA. Data are the means ± SD of triplicate determinations. *P<0.05 and **P<0.01; Student’s *t* test with a two-tailed distribution and two-sample equivalent variance parameters.
(TIF)
