# Supplementary figures and images for: Mitochondria–Nucleus Shuttling FK506-Binding Protein 51 Interacts with TRAF Proteins and Facilitates the RIG-I-Like Receptor-Mediated Expression of Type I IFN
Source: PLoS One. 2014 May 1;9(5):e95992. doi: 10.1371/journal.pone.0095992 (PMC4006813; doi:10.1371/journal.pone.0095992)

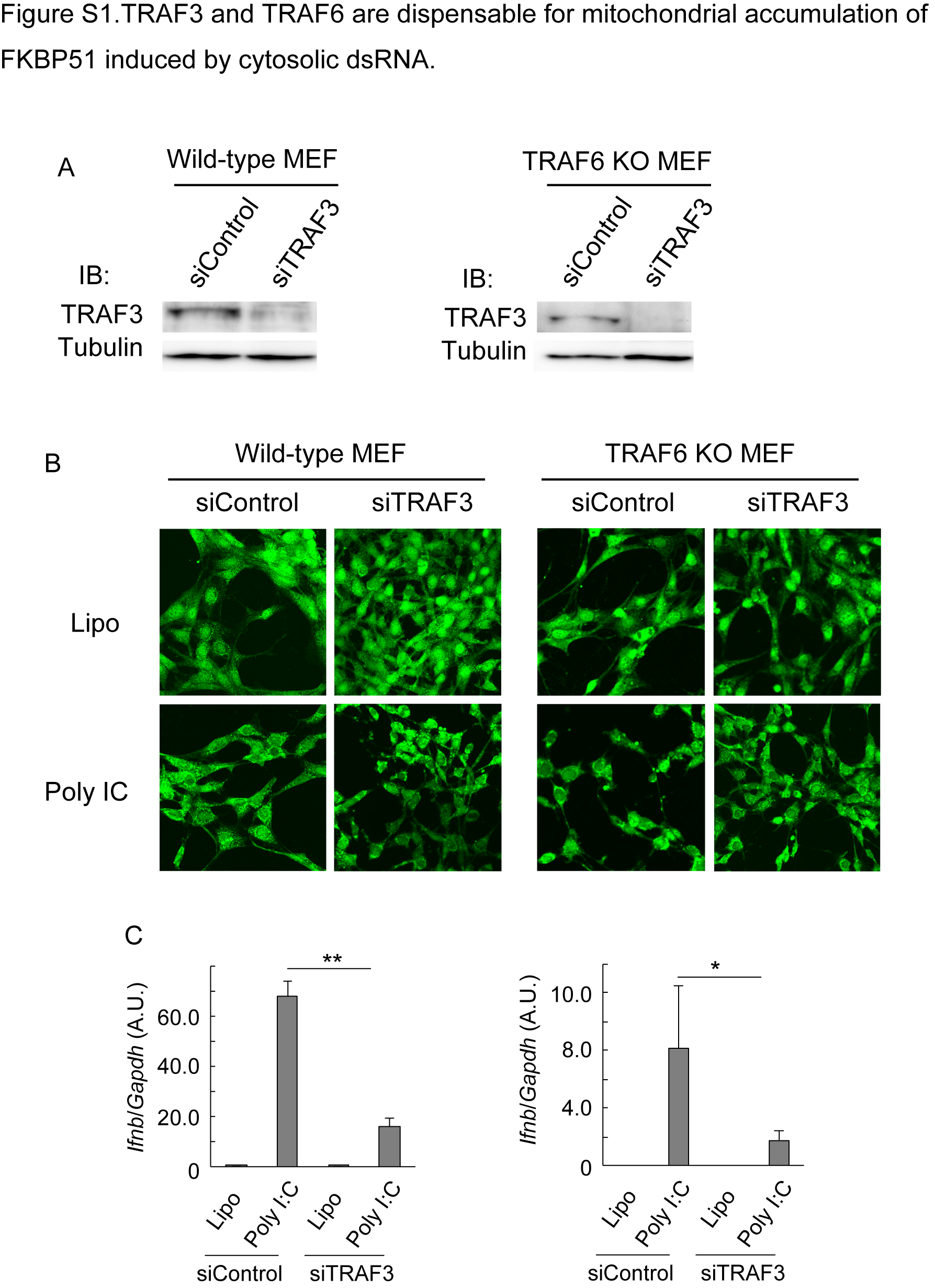

Supplement: Figure S1 — TRAF3 and TRAF6 are dispensable for mitochondrial accumulation of FKBP51 induced by cytosolic dsRNA. (A) Knockdown of TRAF3 in MEF cells. Total cell lysates from TRAF3-knockdown MEF cells (left panel; siTRAF3), control MEF cells (left panel; siControl), TRAF3-knockdown TRAF6-deficient MEF cells (right panel; siTRAF3), and control TRAF6-deficient MEF cells (right panel; siControl) were analyzed with western blotting using anti-TRAF3 antibody (upper panel) or anti-tubulin antibody (lower panel) as the loading control. (B) Cytoplasmic accumulation of FKBP51 in MEF cells after stimulation with cytosolic dsRNA. Wild-type MEF cells, TRAF3-knockdown MEF cells, TRAF6-deficient MEF cells, TRAF3-knockdown TRAF6-deficient MEF cells were stimulated by lipofectamine with or without poly I:C. Endogenous FKBP51 was detected with an anti-FKBP51 antibody. (C) TRAF3- knockdown MEF cells (left figure), control MEF cells (left figure), TRAF3-knockdown TRAF6-deficient MEF cells (right figure) and control TRAF6-deficient MEF cells (right figure) were stimulated by lipofectamine with or without poly I:C. The expression levels of IFNβ was evaluated with a qPCR analysis and normalized to the level of GAPDH mRNA. Data are the means ± SD of triplicate determinations. *P<0.05 and **P<0.01; Student’s t test with a two-tailed distribution and two-sample equivalent variance parameters. (TIF) [file pone.0095992.s001.tif]
